# Bis(2,2′-bipyridine-κ^2^ *N*,*N*′)(thio­cyanato-κ*N*)copper(II) perchlorate

**DOI:** 10.1107/S1600536809031067

**Published:** 2009-08-12

**Authors:** Qian Li, Dong Zhang, Chun-Ling Chen, Lin Yan

**Affiliations:** a Institute of Fine Chemistry and Engineering, College of Chemistry and Chemical Engineering, Henan University, Kaifeng 475004, People’s Republic of China; b Institute of Molecular and Crystal Engineering, College of Chemistry and Chemical Engineering, Henan University, Kaifeng 475004, People’s Republic of China; c Institute of Pharmacy, Henan University, Kaifeng 475004, People’s Republic of China; d Key Laboratory of Natural Medicine and Immunal Engineering, Henan University, Kaifeng 475004, People’s Republic of China

## Abstract

The asymmetric unit of title compound, [Cu(NCS)(C_10_H_8_N_2_)_2_]ClO_4_, contains a bis­(2,2′-bipyridine)(isothio­cyanato)copper(II) cation and a perchlorate anion. In the cation, the Cu^2+^ ion is coordinated by four N atoms from two bidentate 2,2′-bipyridine mol­ecules and an N atom from an isothio­cyanate anion, resulting in a distorted CuN_5_ pyramidal configuration. The crystal structure is stabilized by weak inter­molecular C—H⋯O and C—H⋯S hydrogen bonds, and weak π–π inter­actions between 2,2′-bipyridine rings [centroid–centroid distance = 3.908 (4) Å]. The perchlorate counteranion is disordered over two positions in a 0.66:0.34 ratio.

## Related literature

For the potenial applications of metal-organic coordination compounds in catalysis, non-linear optics, gas absorption, luminescene and magnetism, see: Kitagawa & Matsuda (2007 ▶); Maspoch *et al.* (2007 ▶).
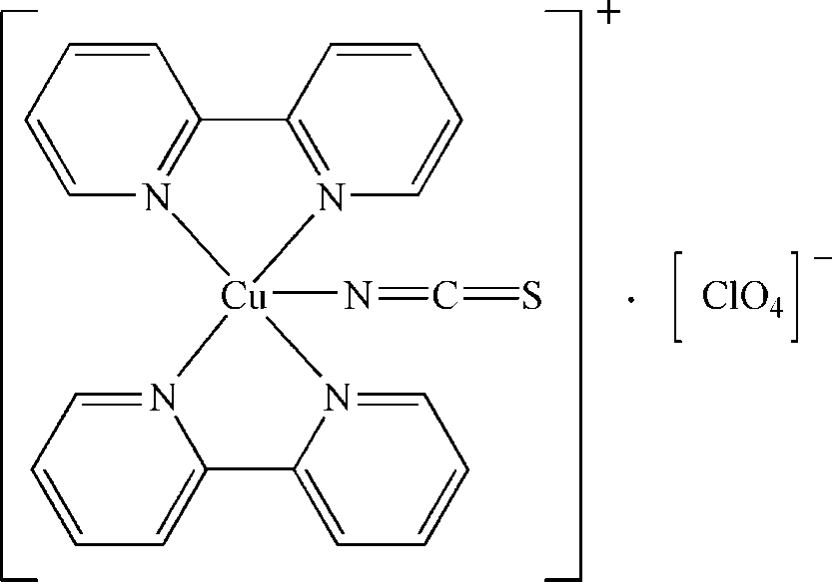


## Experimental

### 

#### Crystal data

[Cu(NCS)(C_10_H_8_N_2_)_2_]ClO_4_
*M* *_r_* = 533.46
Monoclinic, 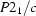

*a* = 15.151 (2) Å
*b* = 8.9518 (13) Å
*c* = 19.0409 (17) Å
β = 120.306 (7)°
*V* = 2229.6 (5) Å^3^
*Z* = 4
Mo *K*α radiation
μ = 1.23 mm^−1^
*T* = 293 K
0.21 × 0.15 × 0.13 mm

#### Data collection

Bruker SMART CCD area-detector diffractometer
Absorption correction: multi-scan (*SADABS*; Sheldrick, 2001 ▶) *T*_min_ = 0.782, *T*_max_ = 0.856
10831 measured reflections
3917 independent reflections
2370 reflections with *I* > 2σ(*I*)
*R*_int_ = 0.043

#### Refinement

*R*[*F* ^2^ > 2σ(*F* ^2^)] = 0.049
*wR*(*F* ^2^) = 0.116
*S* = 1.03
3917 reflections
335 parameters
44 restraints
H-atom parameters constrained
Δρ_max_ = 0.60 e Å^−3^
Δρ_min_ = −0.82 e Å^−3^

### 

Data collection: *SMART* (Bruker, 2001 ▶); cell refinement: *SAINT-Plus* (Bruker, 2001 ▶); data reduction: *SAINT-Plus*; program(s) used to solve structure: *SHELXS97* (Sheldrick, 2008 ▶); program(s) used to refine structure: *SHELXL97* (Sheldrick, 2008 ▶); molecular graphics: *PLATON* (Spek, 2009 ▶); software used to prepare material for publication: *PLATON*.

## Supplementary Material

Crystal structure: contains datablocks global, I. DOI: 10.1107/S1600536809031067/at2856sup1.cif

Structure factors: contains datablocks I. DOI: 10.1107/S1600536809031067/at2856Isup2.hkl

Additional supplementary materials:  crystallographic information; 3D view; checkCIF report

## Figures and Tables

**Table 1:** Selected geometric parameters (Å, °)

Cu1—N5	1.968 (4)
Cu1—N4	1.985 (3)
Cu1—N1	1.992 (4)
Cu1—N2	2.058 (4)
Cu1—N3	2.102 (4)

**Table 2:** Hydrogen-bond geometry (Å, °)

*D*—H⋯*A*	*D*—H	H⋯*A*	*D*⋯*A*	*D*—H⋯*A*
C7—H7*A*⋯O4′^i^	0.93	2.55	3.176 (15)	125
C10—H10*A*⋯S1^ii^	0.93	2.85	3.587 (6)	137
C18—H18*A*⋯O1′^iii^	0.93	2.45	3.335 (13)	159

Symmetry codes: (i) 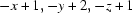; (ii) 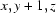; (iii) 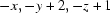.

**Fig. 1.**
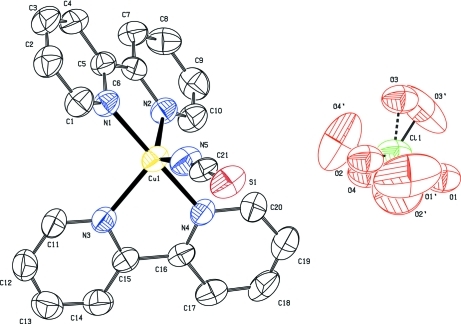
The molecular structure of (I), showing the atom-labelling scheme. Displacement ellipsoids are drawn at the 50% probability level. Hydrogen atoms are omitted for clarity.

## supplementary crystallographic information

### Comment

Recently, more attentions have been paid to metal-organic coordination compounds (MOCPs) due to their potenial applications in catalysis, nonlinear optics, gas absorption, luminescene and magnetism (Maspoch *et al.* 2007, Kitagawa & Matsuda 2007). In the field of coordination chemistry, dual-ligand or multidentate ligands are usually engaged in the costruction of MOCPs, among which N,*N*-bidentate ligands (such as 2,2'-bipyridine) is familiar chelate ligand. Herein, we report the structure of the title compound (I) containing organic dual ligands.

The title compound (I) consists of one [Cu(C_10_H_8_N_2_)_2_(SCN)]^+^ complex cations, one disordered [ClO_4_]^-^ anion (Fig.1). In the molecular structure, the Cu^2+^ centre is coordinated by five N atoms, among which four N atoms come from two bidentate 2,2'-bipyridine molecule and another one N atom from an isothiocyanato anion. The environment of the Cu^2+^ cation is in a distorted pyramidal geometry with Cu–N bond lengths ranging from 1.968 (4) to 2.102 (4) Å (Table 1).

In addition, the crystal structure is stability by weak intermolecular C—H···O and C—H···S hydrogen bonds (Table 2), and weak *π-π* interactions between 2,2'-bipyridine rings with centroid-to centroid distance of 3.908 (4) Å.

### Experimental

2,2'-Bipyridine (1 mol, 0.16 g) was suspended in 20 ml

ethanol solution, to which Cu(ClO_4_)^.^2H_2_O (0.5 mmol, 0.19 g) was added, and then KSCN (0.5 mmol, 0.5 g) were added to the mixture. It was stirred under reflux for 4 h. The solution was cooled and filtered, and the filtrate was kept at the room temperature. After ten days, green blocks of (I) were obtained.

### Refinement

H atoms were treated as riding, with C—H distances of 0.93 Å, and were refined as riding with *U*_iso_(*H*) = 1.2*U*_eq_(C).

### Crystal data

**Table:**

[Cu(NCS)(C_10_H_8_N_2_)_2_]ClO_4_	*F*(000) = 1084
*M**_r_* = 533.46	*D*_x_ = 1.589 Mg m^−^^3^
Monoclinic, *P*2_1_/*c*	Mo *K*α radiation, λ = 0.71073 Å
Hall symbol: -P 2ybc	Cell parameters from 2091 reflections
*a* = 15.151 (2) Å	θ = 2.5–20.5°
*b* = 8.9518 (13) Å	µ = 1.23 mm^−^^1^
*c* = 19.0409 (17) Å	*T* = 293 K
β = 120.306 (7)°	Block, green
*V* = 2229.6 (5) Å^3^	0.21 × 0.15 × 0.13 mm
*Z* = 4	

### Data collection

**Table:**

Bruker SMART CCD area-detector diffractometer	3917 independent reflections
Radiation source: fine-focus sealed tube	2370 reflections with *I* > 2σ(*I*)
graphite	*R*_int_ = 0.043
φ and ω scans	θ_max_ = 25.1°, θ_min_ = 2.2°
Absorption correction: multi-scan (*SADABS*; Sheldrick, 2001)	*h* = −9→18
*T*_min_ = 0.782, *T*_max_ = 0.856	*k* = −10→10
10831 measured reflections	*l* = −22→20

### Refinement

**Table:**

Refinement on *F*^2^	Primary atom site location: structure-invariant direct methods
Least-squares matrix: full	Secondary atom site location: difference Fourier map
*R*[*F*^2^ > 2σ(*F*^2^)] = 0.049	Hydrogen site location: inferred from neighbouring sites
*wR*(*F*^2^) = 0.116	H-atom parameters constrained
*S* = 1.03	*w* = 1/[σ^2^(*F*_o_^2^) + (0.0453*P*)^2^] where *P* = (*F*_o_^2^ + 2*F*_c_^2^)/3
3917 reflections	(Δ/σ)_max_ < 0.001
335 parameters	Δρ_max_ = 0.60 e Å^−^^3^
44 restraints	Δρ_min_ = −0.82 e Å^−^^3^

### Special details

**Table:**

Geometry. All e.s.d.'s (except the e.s.d. in the dihedral angle between two l.s. planes) are estimated using the full covariance matrix. The cell e.s.d.'s are taken into account individually in the estimation of e.s.d.'s in distances, angles and torsion angles; correlations between e.s.d.'s in cell parameters are only used when they are defined by crystal symmetry. An approximate (isotropic) treatment of cell e.s.d.'s is used for estimating e.s.d.'s involving l.s. planes.
Refinement. Refinement of *F*^2^ against ALL reflections. The weighted *R*-factor *wR* and goodness of fit *S* are based on *F*^2^, conventional *R*-factors *R* are based on *F*, with *F* set to zero for negative *F*^2^. The threshold expression of *F*^2^ > σ(*F*^2^) is used only for calculating *R*-factors(gt) *etc*. and is not relevant to the choice of reflections for refinement. *R*-factors based on *F*^2^ are statistically about twice as large as those based on *F*, and *R*- factors based on ALL data will be even larger.

### Fractional atomic coordinates and isotropic or equivalent isotropic displacement parameters (Å^2^)

**Table:**

	*x*	*y*	*z*	*U*_iso_*/*U*_eq_	Occ. (<1)
Cu1	0.28304 (4)	0.52479 (6)	0.58542 (3)	0.0504 (2)	
S1	0.21213 (11)	0.09251 (15)	0.69020 (9)	0.0736 (4)	
N1	0.4011 (3)	0.4415 (4)	0.5796 (2)	0.0489 (10)	
N2	0.3811 (3)	0.7044 (4)	0.6288 (2)	0.0478 (9)	
N3	0.1728 (3)	0.5378 (4)	0.4610 (2)	0.0475 (9)	
N4	0.1701 (3)	0.6262 (4)	0.5907 (2)	0.0488 (9)	
N5	0.2680 (3)	0.3429 (5)	0.6365 (3)	0.0659 (12)	
C1	0.4060 (4)	0.3036 (5)	0.5556 (3)	0.0588 (13)	
H1A	0.3494	0.2416	0.5375	0.071*	
C2	0.4912 (4)	0.2495 (6)	0.5565 (3)	0.0637 (14)	
H2A	0.4918	0.1535	0.5381	0.076*	
C3	0.5754 (4)	0.3400 (6)	0.5852 (3)	0.0657 (14)	
H3A	0.6348	0.3055	0.5875	0.079*	
C4	0.5712 (4)	0.4814 (5)	0.6103 (3)	0.0576 (13)	
H4A	0.6278	0.5439	0.6300	0.069*	
C5	0.4828 (3)	0.5312 (5)	0.6062 (3)	0.0453 (11)	
C6	0.4705 (3)	0.6823 (5)	0.6308 (3)	0.0456 (11)	
C7	0.5427 (4)	0.7926 (5)	0.6539 (3)	0.0552 (13)	
H7A	0.6031	0.7753	0.6537	0.066*	
C8	0.5246 (4)	0.9294 (6)	0.6772 (3)	0.0651 (14)	
H8A	0.5732	1.0050	0.6941	0.078*	
C9	0.4346 (4)	0.9526 (5)	0.6753 (3)	0.0666 (14)	
H9A	0.4209	1.0440	0.6911	0.080*	
C10	0.3643 (4)	0.8385 (5)	0.6498 (3)	0.0584 (13)	
H10A	0.3021	0.8559	0.6471	0.070*	
C11	0.1745 (4)	0.4783 (5)	0.3972 (3)	0.0570 (13)	
H11A	0.2310	0.4217	0.4067	0.068*	
C12	0.0976 (4)	0.4968 (5)	0.3191 (3)	0.0642 (14)	
H12A	0.1007	0.4517	0.2764	0.077*	
C13	0.0156 (4)	0.5828 (6)	0.3046 (3)	0.0702 (15)	
H13A	−0.0374	0.5987	0.2516	0.084*	
C14	0.0126 (4)	0.6453 (5)	0.3689 (3)	0.0617 (14)	
H14A	−0.0424	0.7049	0.3600	0.074*	
C15	0.0910 (3)	0.6196 (5)	0.4464 (3)	0.0462 (11)	
C16	0.0909 (3)	0.6708 (5)	0.5202 (3)	0.0467 (11)	
C17	0.0122 (4)	0.7529 (6)	0.5185 (3)	0.0660 (15)	
H17A	−0.0420	0.7867	0.4694	0.079*	
C18	0.0157 (4)	0.7834 (6)	0.5909 (4)	0.0721 (16)	
H18A	−0.0365	0.8384	0.5908	0.087*	
C19	0.0945 (4)	0.7339 (5)	0.6616 (4)	0.0675 (15)	
H19A	0.0970	0.7527	0.7107	0.081*	
C20	0.1706 (4)	0.6553 (5)	0.6597 (3)	0.0612 (14)	
H20A	0.2250	0.6206	0.7085	0.073*	
C21	0.2448 (3)	0.2379 (5)	0.6594 (3)	0.0514 (12)	
Cl1	0.24431 (10)	0.98729 (15)	0.40224 (9)	0.0726 (4)	
O1'	0.1951 (6)	1.1109 (9)	0.4087 (6)	0.190 (5)	0.66
O2'	0.1768 (7)	0.8670 (8)	0.3661 (5)	0.193 (5)	0.66
O3'	0.3286 (6)	0.9451 (11)	0.4777 (4)	0.177 (5)	0.66
O4'	0.2788 (8)	1.0206 (15)	0.3457 (6)	0.268 (7)	0.66
O1	0.1870 (7)	0.9781 (12)	0.4423 (6)	0.0755 (10)	0.34
O2	0.2143 (8)	0.8817 (10)	0.3404 (6)	0.0728 (10)	0.34
O3	0.3518 (6)	0.9693 (14)	0.4598 (7)	0.0730 (10)	0.34
O4	0.2356 (9)	1.1359 (8)	0.3709 (6)	0.0730 (10)	0.34

### Atomic displacement parameters (Å^2^)

**Table:**

	*U*^11^	*U*^22^	*U*^33^	*U*^12^	*U*^13^	*U*^23^
Cu1	0.0461 (4)	0.0518 (4)	0.0585 (4)	0.0054 (3)	0.0303 (3)	0.0020 (3)
S1	0.0721 (10)	0.0594 (9)	0.1024 (12)	0.0078 (7)	0.0536 (9)	0.0197 (8)
N1	0.049 (2)	0.045 (2)	0.057 (2)	0.0044 (19)	0.030 (2)	−0.0022 (19)
N2	0.047 (2)	0.043 (2)	0.056 (3)	0.0038 (18)	0.028 (2)	−0.0002 (19)
N3	0.046 (2)	0.052 (2)	0.051 (2)	0.0014 (19)	0.029 (2)	0.0017 (19)
N4	0.047 (2)	0.054 (2)	0.054 (3)	0.0036 (19)	0.032 (2)	0.001 (2)
N5	0.058 (3)	0.068 (3)	0.077 (3)	0.003 (2)	0.038 (3)	0.009 (2)
C1	0.063 (3)	0.048 (3)	0.071 (4)	0.004 (3)	0.038 (3)	−0.003 (3)
C2	0.071 (4)	0.054 (3)	0.074 (4)	0.018 (3)	0.042 (3)	0.001 (3)
C3	0.057 (4)	0.073 (4)	0.075 (4)	0.020 (3)	0.039 (3)	0.002 (3)
C4	0.043 (3)	0.066 (3)	0.064 (3)	0.006 (2)	0.027 (3)	0.000 (3)
C5	0.040 (3)	0.049 (3)	0.047 (3)	0.004 (2)	0.022 (2)	0.003 (2)
C6	0.041 (3)	0.051 (3)	0.039 (3)	0.004 (2)	0.016 (2)	0.004 (2)
C7	0.042 (3)	0.058 (3)	0.063 (3)	−0.003 (2)	0.024 (3)	−0.003 (3)
C8	0.052 (3)	0.056 (3)	0.075 (4)	−0.007 (3)	0.023 (3)	−0.003 (3)
C9	0.077 (4)	0.050 (3)	0.065 (4)	0.005 (3)	0.031 (3)	−0.009 (3)
C10	0.056 (3)	0.053 (3)	0.071 (4)	0.006 (3)	0.036 (3)	−0.002 (3)
C11	0.059 (3)	0.065 (3)	0.061 (3)	0.002 (3)	0.040 (3)	0.000 (3)
C12	0.070 (4)	0.078 (4)	0.057 (4)	−0.012 (3)	0.041 (3)	−0.008 (3)
C13	0.056 (4)	0.090 (4)	0.057 (4)	−0.013 (3)	0.022 (3)	0.007 (3)
C14	0.048 (3)	0.072 (4)	0.061 (4)	0.005 (3)	0.024 (3)	0.006 (3)
C15	0.044 (3)	0.042 (3)	0.056 (3)	−0.001 (2)	0.028 (3)	0.002 (2)
C16	0.037 (3)	0.047 (3)	0.058 (3)	−0.001 (2)	0.026 (3)	−0.002 (2)
C17	0.049 (3)	0.072 (4)	0.075 (4)	0.013 (3)	0.030 (3)	0.004 (3)
C18	0.063 (4)	0.070 (4)	0.103 (5)	0.004 (3)	0.057 (4)	−0.014 (4)
C19	0.075 (4)	0.065 (4)	0.085 (4)	−0.009 (3)	0.057 (4)	−0.013 (3)
C20	0.063 (3)	0.069 (3)	0.060 (3)	0.000 (3)	0.037 (3)	−0.002 (3)
C21	0.040 (3)	0.053 (3)	0.064 (3)	0.010 (2)	0.029 (3)	0.001 (3)
Cl1	0.0573 (8)	0.0714 (9)	0.0791 (10)	0.0056 (7)	0.0270 (8)	−0.0106 (7)
O1'	0.128 (7)	0.134 (7)	0.300 (13)	0.047 (6)	0.102 (8)	−0.069 (7)
O2'	0.201 (10)	0.176 (8)	0.174 (9)	−0.140 (8)	0.072 (7)	−0.028 (7)
O3'	0.183 (9)	0.215 (10)	0.062 (5)	0.082 (8)	0.008 (6)	0.001 (5)
O4'	0.224 (13)	0.41 (2)	0.259 (14)	−0.040 (12)	0.189 (13)	0.037 (12)
O1	0.0592 (14)	0.0735 (14)	0.0817 (15)	0.0063 (13)	0.0267 (13)	−0.0106 (13)
O2	0.0582 (14)	0.0710 (14)	0.0786 (15)	0.0059 (13)	0.0266 (13)	−0.0119 (13)
O3	0.0569 (13)	0.0713 (14)	0.0797 (15)	0.0063 (13)	0.0263 (13)	−0.0118 (13)
O4	0.0577 (13)	0.0715 (14)	0.0796 (15)	0.0058 (13)	0.0270 (13)	−0.0097 (13)

### Geometric parameters (Å, °)

**Table:**

Cu1—N5	1.968 (4)	C11—C12	1.360 (7)
Cu1—N4	1.985 (3)	C11—H11A	0.9300
Cu1—N1	1.992 (4)	C12—C13	1.365 (6)
Cu1—N2	2.058 (4)	C12—H12A	0.9300
Cu1—N3	2.102 (4)	C13—C14	1.369 (6)
S1—C21	1.605 (5)	C13—H13A	0.9300
N1—C1	1.331 (5)	C14—C15	1.368 (6)
N1—C5	1.342 (5)	C14—H14A	0.9300
N2—C10	1.331 (5)	C15—C16	1.478 (6)
N2—C6	1.349 (5)	C16—C17	1.387 (6)
N3—C11	1.339 (5)	C17—C18	1.380 (7)
N3—C15	1.342 (5)	C17—H17A	0.9300
N4—C16	1.332 (5)	C18—C19	1.347 (7)
N4—C20	1.335 (5)	C18—H18A	0.9300
N5—C21	1.163 (5)	C19—C20	1.368 (6)
C1—C2	1.371 (6)	C19—H19A	0.9300
C1—H1A	0.9300	C20—H20A	0.9300
C2—C3	1.371 (6)	Cl1—O1'	1.374 (5)
C2—H2A	0.9300	Cl1—O2	1.395 (7)
C3—C4	1.366 (6)	Cl1—O2'	1.403 (5)
C3—H3A	0.9300	Cl1—O3'	1.409 (5)
C4—C5	1.376 (6)	Cl1—O1	1.419 (6)
C4—H4A	0.9300	Cl1—O4	1.437 (7)
C5—C6	1.473 (6)	Cl1—O3	1.442 (7)
C6—C7	1.372 (6)	Cl1—O4'	1.446 (6)
C7—C8	1.377 (6)	O1'—O4	1.180 (11)
C7—H7A	0.9300	O1'—O1	1.385 (11)
C8—C9	1.362 (7)	O2'—O2	0.927 (11)
C8—H8A	0.9300	O2'—O1	1.701 (11)
C9—C10	1.375 (6)	O3'—O3	0.638 (17)
C9—H9A	0.9300	O4'—O4	1.429 (12)
C10—H10A	0.9300	O4'—O2	1.553 (12)
			
N5—Cu1—N4	92.02 (16)	N3—C15—C16	114.5 (4)
N5—Cu1—N1	92.83 (16)	C14—C15—C16	123.8 (4)
N4—Cu1—N1	174.75 (15)	N4—C16—C17	120.5 (4)
N5—Cu1—N2	133.48 (16)	N4—C16—C15	115.7 (4)
N4—Cu1—N2	95.11 (14)	C17—C16—C15	123.7 (5)
N1—Cu1—N2	80.08 (15)	C18—C17—C16	118.9 (5)
N5—Cu1—N3	112.25 (16)	C18—C17—H17A	120.6
N4—Cu1—N3	79.43 (15)	C16—C17—H17A	120.6
N1—Cu1—N3	100.55 (14)	C19—C18—C17	120.1 (5)
N2—Cu1—N3	114.25 (14)	C19—C18—H18A	119.9
C1—N1—C5	119.0 (4)	C17—C18—H18A	119.9
C1—N1—Cu1	125.0 (3)	C18—C19—C20	118.5 (5)
C5—N1—Cu1	116.0 (3)	C18—C19—H19A	120.8
C10—N2—C6	117.8 (4)	C20—C19—H19A	120.8
C10—N2—Cu1	128.0 (3)	N4—C20—C19	122.7 (5)
C6—N2—Cu1	114.1 (3)	N4—C20—H20A	118.7
C11—N3—C15	117.8 (4)	C19—C20—H20A	118.7
C11—N3—Cu1	129.2 (3)	N5—C21—S1	179.5 (6)
C15—N3—Cu1	112.9 (3)	O1'—Cl1—O2	131.4 (6)
C16—N4—C20	119.3 (4)	O1'—Cl1—O2'	111.6 (5)
C16—N4—Cu1	116.5 (3)	O2—Cl1—O2'	38.7 (5)
C20—N4—Cu1	124.2 (3)	O1'—Cl1—O3'	112.2 (5)
C21—N5—Cu1	170.6 (4)	O2—Cl1—O3'	114.9 (6)
N1—C1—C2	122.6 (5)	O2'—Cl1—O3'	110.8 (5)
N1—C1—H1A	118.7	O1'—Cl1—O1	59.4 (5)
C2—C1—H1A	118.7	O2—Cl1—O1	112.8 (6)
C1—C2—C3	118.5 (5)	O2'—Cl1—O1	74.1 (5)
C1—C2—H2A	120.7	O3'—Cl1—O1	85.6 (5)
C3—C2—H2A	120.7	O1'—Cl1—O4	49.6 (5)
C4—C3—C2	119.2 (5)	O2—Cl1—O4	110.7 (6)
C4—C3—H3A	120.4	O2'—Cl1—O4	128.0 (6)
C2—C3—H3A	120.4	O3'—Cl1—O4	121.2 (6)
C3—C4—C5	119.8 (5)	O1—Cl1—O4	108.9 (5)
C3—C4—H4A	120.1	O1'—Cl1—O3	118.5 (7)
C5—C4—H4A	120.1	O2—Cl1—O3	109.2 (6)
N1—C5—C4	120.9 (4)	O2'—Cl1—O3	123.4 (7)
N1—C5—C6	115.4 (4)	O3'—Cl1—O3	25.8 (7)
C4—C5—C6	123.8 (4)	O1—Cl1—O3	110.4 (6)
N2—C6—C7	122.0 (4)	O4—Cl1—O3	104.5 (6)
N2—C6—C5	114.3 (4)	O1'—Cl1—O4'	108.5 (5)
C7—C6—C5	123.6 (4)	O2—Cl1—O4'	66.3 (6)
C6—C7—C8	119.2 (4)	O2'—Cl1—O4'	104.0 (5)
C6—C7—H7A	120.4	O3'—Cl1—O4'	109.4 (5)
C8—C7—H7A	120.4	O1—Cl1—O4'	164.2 (6)
C9—C8—C7	119.1 (5)	O4—Cl1—O4'	59.4 (5)
C9—C8—H8A	120.4	O3—Cl1—O4'	84.0 (7)
C7—C8—H8A	120.4	O4—O1'—Cl1	68.0 (4)
C8—C9—C10	118.8 (5)	O4—O1'—O1	129.8 (6)
C8—C9—H9A	120.6	Cl1—O1'—O1	61.9 (4)
C10—C9—H9A	120.6	O2—O2'—Cl1	70.2 (6)
N2—C10—C9	123.0 (5)	O2—O2'—O1	123.5 (7)
N2—C10—H10A	118.5	Cl1—O2'—O1	53.4 (3)
C9—C10—H10A	118.5	O3—O3'—Cl1	80.0 (9)
N3—C11—C12	123.1 (5)	O4—O4'—Cl1	60.0 (4)
N3—C11—H11A	118.4	O4—O4'—O2	102.7 (6)
C12—C11—H11A	118.4	Cl1—O4'—O2	55.3 (4)
C11—C12—C13	118.7 (5)	O1'—O1—Cl1	58.7 (4)
C11—C12—H12A	120.7	O1'—O1—O2'	95.7 (6)
C13—C12—H12A	120.7	Cl1—O1—O2'	52.5 (3)
C12—C13—C14	119.2 (5)	O2'—O2—Cl1	71.1 (6)
C12—C13—H13A	120.4	O2'—O2—O4'	127.8 (8)
C14—C13—H13A	120.4	Cl1—O2—O4'	58.4 (4)
C15—C14—C13	119.6 (5)	O3'—O3—Cl1	74.2 (9)
C15—C14—H14A	120.2	O1'—O4—O4'	122.4 (7)
C13—C14—H14A	120.2	O1'—O4—Cl1	62.4 (4)
N3—C15—C14	121.6 (4)	O4'—O4—Cl1	60.6 (4)

### Hydrogen-bond geometry (Å, °)

**Table:**

*D*—H···*A*	*D*—H	H···*A*	*D*···*A*	*D*—H···*A*
C7—H7A···O4'^i^	0.93	2.55	3.176 (15)	125
C10—H10A···S1^ii^	0.93	2.85	3.587 (6)	137
C18—H18A···O1'^iii^	0.93	2.45	3.335 (13)	159

Symmetry codes: (i) −*x*+1, −*y*+2, −*z*+1; (ii) *x*, *y*+1, *z*; (iii) −*x*, −*y*+2, −*z*+1.
